# Interferon-γ Induces Immunoproteasomes and the Presentation of MHC I-Associated Peptides on Human Salivary Gland Cells

**DOI:** 10.1371/journal.pone.0102878

**Published:** 2014-08-07

**Authors:** Martha E. Arellano-Garcia, Kaori Misuno, Simon D. Tran, Shen Hu

**Affiliations:** 1 School of Dentistry and Jonsson Comprehensive Cancer Center, University of California Los Angeles, Los Angeles, California, United States of America; 2 Faculty of Dentistry, McGill University, Montreal, Quebec, Canada; Leiden University Medical Center, Netherlands

## Abstract

A prominent histopathological feature of Sjögren's syndrome, an autoimmune disease, is the presence of lymphocytic infiltrates in the salivary and lachrymal glands. Such infiltrates are comprised of activated lymphocytes and macrophages, and known to produce multiple cytokines including interferon-gamma (IFN-γ). In this study, we have demonstrated that IFN-γ strongly induces the expression of immunoproteasome beta subunits (β1i, β2i and β5i) and immunoproteasome activity but conversely inhibits the expression of proteasome beta subunits (β1, β2 and β5) in human salivary gland (HSG) cells. Mass spectrometric analysis has revealed potential MHC I-associated peptides on the HSG cells, including a tryptic peptide derived from salivary amylase, due to IFN-γ stimulation. These results suggest that IFN-γ induces immunoproteasomes in HSG cells, leading to enhanced presentation of MHC I-associated peptides on cell surface. These peptide-presenting salivary gland cells may be recognized and targeted by auto-reactive T lymphocytes. We have also found that lactacystin, a proteasome inhibitor, inhibits the expression of β1 subunit in HSG cells and blocks the IFN-γ-induced expression of β1i and immunoproteasome activity. However, the expression of β2i and β5i in HSG cells is not affected by lactacystin. These results may add new insight into the mechanism regarding how lactacystin blocks the action of proteasomes or immunoproteasomes.

## Introduction

Proteasomes are large protein complexes that function to degrade a wide spectrum of proteins involved in the regulation of cellular processes. The constitutive proteasome is a cylindrical structure consisting of four stacked rings and two caps [1], [2]. The two inner rings are composed of seven beta (β) subunits containing active protease-like sites. These proteolytic sites are located on the interior surface of the rings, so that the target protein must enter the central core for degradation. The two outer rings each consist of 7 alpha subunits, which assemble a gating channel for proteins to enter the proteasome “core”. Formation of the 26S proteasome also requires the addition of a regulatory cap structure to each end of the four stacked rings. These caps recognize ubiquitinylated proteins, unfold these degradation substrates and thread them into the inner chamber of the proteasome complex where proteolysis takes place.

In regular proteasomes, the β1, β2, and β5 subunits mediate caspase-like, trypsin-like, and chymotrypsin-like activities, respectively. These β subunits may be replaced by their βi counterparts (β1i, β2i and β5i) in some cells when they are treated with interferon-gamma (IFN-γ), leading to the formation of immunoproteasomes. Compared to regular proteasomes, immunoproteasomes exhibit higher trypsin-like and chymotrypsin-like activities and a lower caspase-like activity [3]–[5]. Peptides produced by immunoproteasomes mainly contain hydrophobic or basic carboxyl termini which appear to be more efficient at binding to MHC class I. Consequently immunoproteasomes are believed to enhance the generation of antigenic peptides for MHC Class I presentation [6]–[8].

Sjögren's syndrome (SS) is a chronic, autoimmune disease causing dry mouth and eyes in ∼4 million Americans [9], [10]. A prominent histopathological feature of SS is the presence of lymphocytic infiltrates in the salivary and lachrymal glands. Such infiltrates are comprised of activated lymphocytes and macrophages, and known to produce multiple cytokines including IFN-γ. IFN-γ plays an important role in the pathogenesis of SS as evidenced by previous studies. First, the salivary glands of SS patients are infiltrated with massive amount of T lymphocytes. These infiltrated T cells produce significant high levels of IFN-γ [11], [12]. Second, constant stimulation of salivary gland cells with IFN-γ (1000 U/ml) has been shown to induce apoptosis of HSG cells via up-regulation of Fas [13]–[15]. Third, animal model studies suggested that IFN-γ plays a critical role not only during the later immune phase of SS, but also in the early pre-immune phase, independent of effector functions of immune cells. Compared to non-obese diabetic (NOD) mice who develop SS-like symptoms, both IFN-γ and IFN-γ receptor (IFN-γR) gene knockout NOD mice (NOD.IFN-γ^-/-^ & NOD.IFN-γR^-/-^) showed no subsequent autoimmune response against the salivary glands [16]. Last, Ro60 peptide immunization in the abdominal area of female Balb/c mice led to increased levels of IFN-γ and IL-12 systemically and locally in the salivary glands. This implies that the mechanism of action of Ro60 peptide immunization appears to involve an increase in Th1 cytokines, resulting in the induction of salivary gland dysfunction [17].

There have been few studies on the possible role of immunoproteasome in SS pathogenesis [18]–[20]. In both infiltrating and peripheral immune cells, β1i expression was found to be down-regulated in SS patients compared to healthy controls [18], [19]. On the other hand, β5i (LMP7) was found to be over-expressed in the salivary gland epithelial cells of SS patients and therefore suggested as a specific biomarker for SS diagnosis [20]. However, these studies did not reveal the MHC-associated peptides presented on human salivary gland cells due to IFN-γ-mediated activation of immunoproteasomes in SS.

In this study, we have demonstrated that IFN-γ induces the expression of immunoproteasome β subunits but inhibits the expression of constitutive β subunits in HSG cells. Immunoproteasome activity was found to be elevated in the IFN-γ-treated HSG cells, leading to the presentation of MHC I-associated peptides on the cells. We have also shown that lactacystin, a proteasome inhibitor, inhibits the expression of β1i and β1 subunits and therefore blocks the IFN-γ-mediated immunoproteasome activity in HSG cells.

## Results

### IFN-γ induces the expression of β1i, β2i and β5i in HSG cells

**Figure 1:**
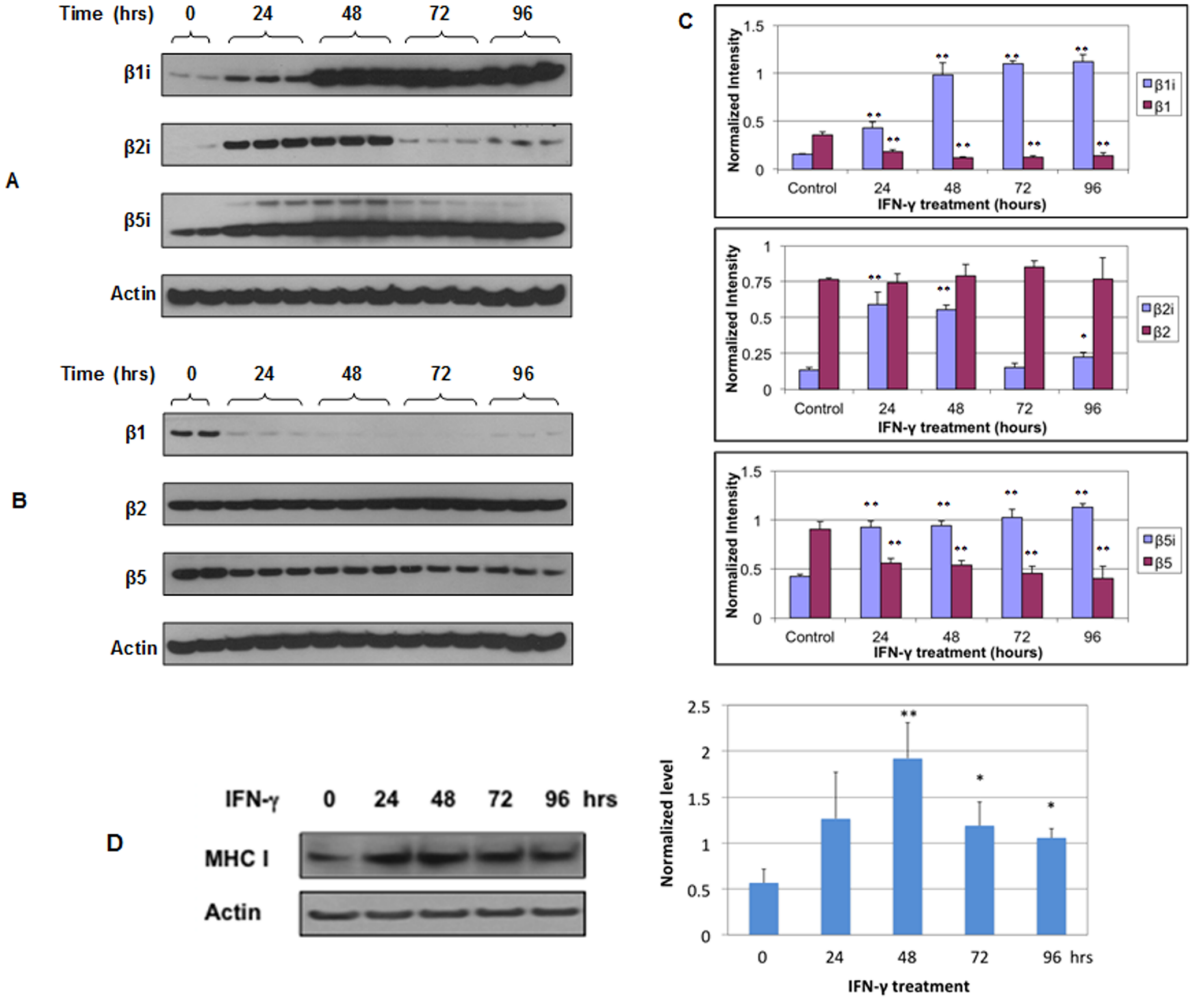
IFN-γ induces the expression of immunoproteasome β subunits in HSG cells. The cells were treated with IFN-γ at 250 U/ml for 24, 48, 72 or 96 hours. Western blotting was used to determine the levels of β1i, β2i and β5i (A) and proteasome β subunits β1, β2 and β5 (B) in IFN-γ-treated cells (n = 3 at each time point) and untreated cells (control, n = 2). (C) Normalized levels of immunoproteasome β subunits in relation to constitutive proteasome β subunits in IFN-γ-treated and untreated HSG cells. For each time point, three biological replicates (three cell lysates samples obtained from three separate wells of the 6-well plate) were used for Western blot analysis and the error bars represent the standard deviations at each time point. Computation of p values was done at each time point versus the control (untreated cells). *, p<0.05; **, p<0.01. (D) Western blot analysis of MHC class I in untreated and IFN-γ-treated HSG cells (250 U/ml for 24–96 hrs). *, p<0.05; **, p<0.01. Similarly, three biological replicates at each experimental condition were used for the analysis.

To investigate the effect of IFN-γ on the expression of immunoproteasome beta subunits β1i, β2i and β5i, we treated HSG cells with IFN-γ (250 Units/ml) for 24, 48, 72 and 96 hours and then compared the expression of β1i, β2i & β5i between untreated and IFN-γ-treated HSG cells with Western blotting. As shown in **Figure 1A**, IFN-γ induced the expression of all three β subunits in HSG cells. Compared to untreated HSG cells, β1i expression increased 2.7-, 6-, 6.9- or 7-fold (p<0.009) while β5i expression increased 2.1-, 2.2-, 2.4- or 2.6-fold (p<0.002), respectively, in HSG cells when treated with IFN-γ for 24, 48, 72, and 96 hours. Significant induction of β2i expression was also observed when HSG cells were treated with IFN-γ for 24 hours (4.4-fold) and 48 hours (4.1-fold). However, further treatment with IFN-γ for 72 and 96 hours resulted in significant decrease of β2i expression in HSG cells.

### IFN-γ inhibits the expression of β1 and β5 in HSG cells

We also investigated the effect of IFN-γ treatment on the expression of constitutive β subunits, β1, β2 and β5 (**Figure 1B**). Compared to untreated HSG cells, β1 expression decreased 2.0-, 3.0-, 2.9-, or 2.5-fold (p<0.002) while β5 expression decreased 1.6-, 1.7-, 2.0-, or 2.2-fold (p<0.01), respectively, when HSG cells were treated with IFN-γ for 24, 48, 72 and 96 hours. The expression of β2 in HSG cells was not significantly altered by IFN-γ treatment.

### IFN-γ up-regulates MHC I in HSG cells

**Figure 1D** shows the Western blot analysis of MHC class I in untreated and IFN-γ-treated HSG cells. IFN-γ treatment significantly up-regulated the expression of MHC class I but not the expression of MHC class II (data not shown) in HSG cells.

### Identification of MHC I-associated peptides

In order to identify MHC I-associated peptides on untreated and IFN-γ-treated HSG cells, we used Co-IP to pull down the MHC class I complex from HSG cells and then separated the peptides from proteins within the MHC class I complex. The isolated proteins were further digested with trypsin and the resulting peptides were identified using LC-MS/MS and database searching. MHC class I was identified from IFN-γ-treated HSG cells, with five peptides matched, and from untreated HSG cells with 4 peptides matched. However, B2M was only identified from IFN-γ-treated HSG cells, with two unique peptides matched (**Table 1**).

**Table 1:** LC-MS/MS identification of tryptic peptides from MHC class I or beta-2-microglobulin.

	Peptides identified by LC-MS/MS	
Protein name	IFN-γ-treated cells	Untreated cells
MHC class I antigen	FIAVGYVDDT QFVR	FIAVGYVDDT QFVR
	FIAVGYVDDT QFVR	FIAVGYVDDT QFVR
	FIAVGYVDDT QFVR	EAGSHTIQMMYGCDVGPDGR YGCDVGPDGR
	APWIEQEGPE YWDR	AYLEGTCVEW LR
	AYLEGTCVEW LR	AYLEGTCVEW LR
	AYLEGTCVEW LR	DGEDQTQDTE LVETRPAGDGTFQK
	DGEDQTQDTE LVETRPAGDGTFQK	
Beta-2-microglobulin	SNFLNCYVSG FHPSDIEVDLLK	Not detected
	SNFLNCYVSG FHPSDIEVDLLK	
	VNHVTLSQPK	
	VNHVTLSQPK	

Note: Redundant peptides are listed in the table because the peptide was detected two or more times.

**Figure 2:**
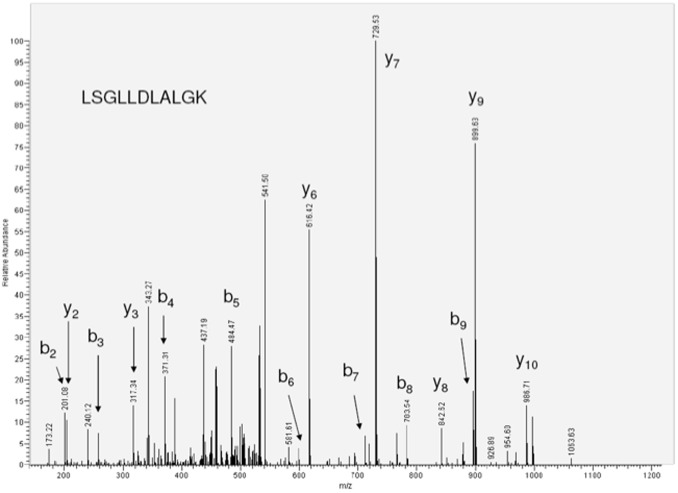
Tandem MS spectrum of MHC I-associated peptide, LSGLLDLALGK, originated from salivary amylase.

The isolated MHC I-associated peptides were directly analyzed by LC-MS/MS (**Table 2**). Five MHC I-associated peptides were identified from the IFN-γ-treated HSG cells whereas none identified from untreated HSG cells. The tandem MS spectrum of LSGLLDLALGK, which is an MHC class I peptide originated from salivary amylase is shown in **Figure 2**. These results imply that IFN-γ induces the expression of immunoproteasomes, leading to the presentation of potential MHC I-associated peptides on HSG cells.

**Table 2:** Potential MHC I-associated peptides on IFN-γ-treated cells identified by LC-MS/MS.

Protein name	MHC I peptides identified
Alpha amylase, salivary	LSGLLDLALGK
Glyceraldehyde-3-phosphate dehydrogenase	QASEGPLK
Splicing factor, arginine/serine-rich 130	LLTEILLDVTDEEIYY
Uncharacterized membrane protein C6orf72	NIMVVGITGAAVVI
Type II cytokeratin-1	SLDLDSIIAEVK

### Lactacystin inhibits IFN-γ-induced expression of β1i subunit in HSG cells

**Figure 3:**
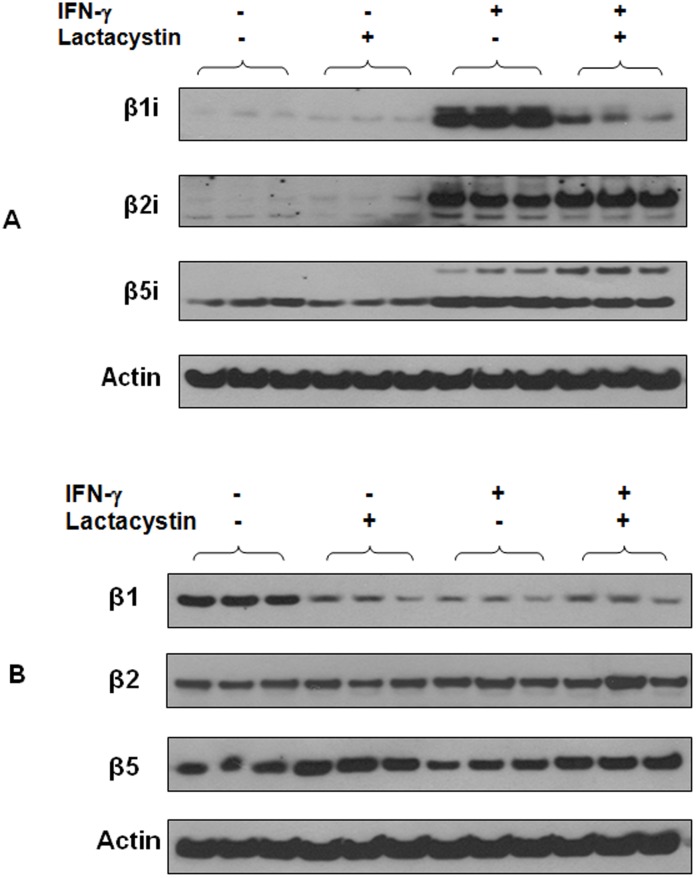
Effect of lactacystin pre-treatment on the expression of β1i, β2i, β5i, β1, β2 and β5 in HSG cells. (A) Western blot analysis of β1i, β2i and β5i in HSG cells (n = 3 per group). (B) Western blot analysis of β1, β2 and β5 in HSG cells (n = 3 per group). At each experimental condition, three cell lysates samples obtained from three separate wells of the 6-well plate were analyzed.

To investigate if a proteasome inhibitor, lactacystin, suppresses IFN-γ-induced expression of immunoproteasome β subunits, we pre-treated HSG cells with lactacystin followed by IFN-γ treatment and then analyzed the expression of βi subunits in the cells. As shown in **Figure 3A**, lactacystin did not significantly affect the endogenous expression of β1i, β2i and β5i in HSG cells. However, lactacystin pretreatment significantly inhibited the expression of both precursor and mature forms of β1i in IFN-γ-treated HSG cells (p<0.004). The expression levels of β2i (p = 0.19) and β5i (p = 0.09) in IFN-γ-treated cells were not significantly altered by lactacystin pre-treatment.

### Lactacystin inhibits the expression of β1 in HSG cells

We also investigated the effect of lactacystin pre-treatment on the expression of constitutive proteasome β subunits in HSG cells (**Figure 3B**). Lactacystin significantly inhibited the expression of β1 (2.7-fold, p = 0.003), slightly up-regulated the expression of β5 (1.5-fold, p = 0.04) but did not affect the expression of β2 in HSG cells (lactacystin-treated versus untreated HSG cells). However, when we compared HSG cells treated with both lactacystin and IFN-γ to those cells treated with IFN-γ only, lactacystin pre-treatment did not significantly alter the expression of β1 (1.2-fold, p = 0.09) and β2 (1.1-fold, p = 0.39) but slightly up-regulated the expression of β5 (1.4-fold, p = 0.006) in the IFN-γ-treated HSG cells.

On the other hand, when we compared HSG cells treated with both lactacystin and IFN-γ to those cells treated with lactacystin only, the expression levels of β1 (1.07-fold, p = 0.75), β2 (1.2-fold, p = 0.26) and β5 (0.88-fold, p = 0.33) in lactacystin-treated HSG cells were not significantly affected by subsequent IFN-γ stimulation.

### Lactacystin inhibits IFN-γ-induced immunoproteasome activities in HSG cells

**Figure 4:**
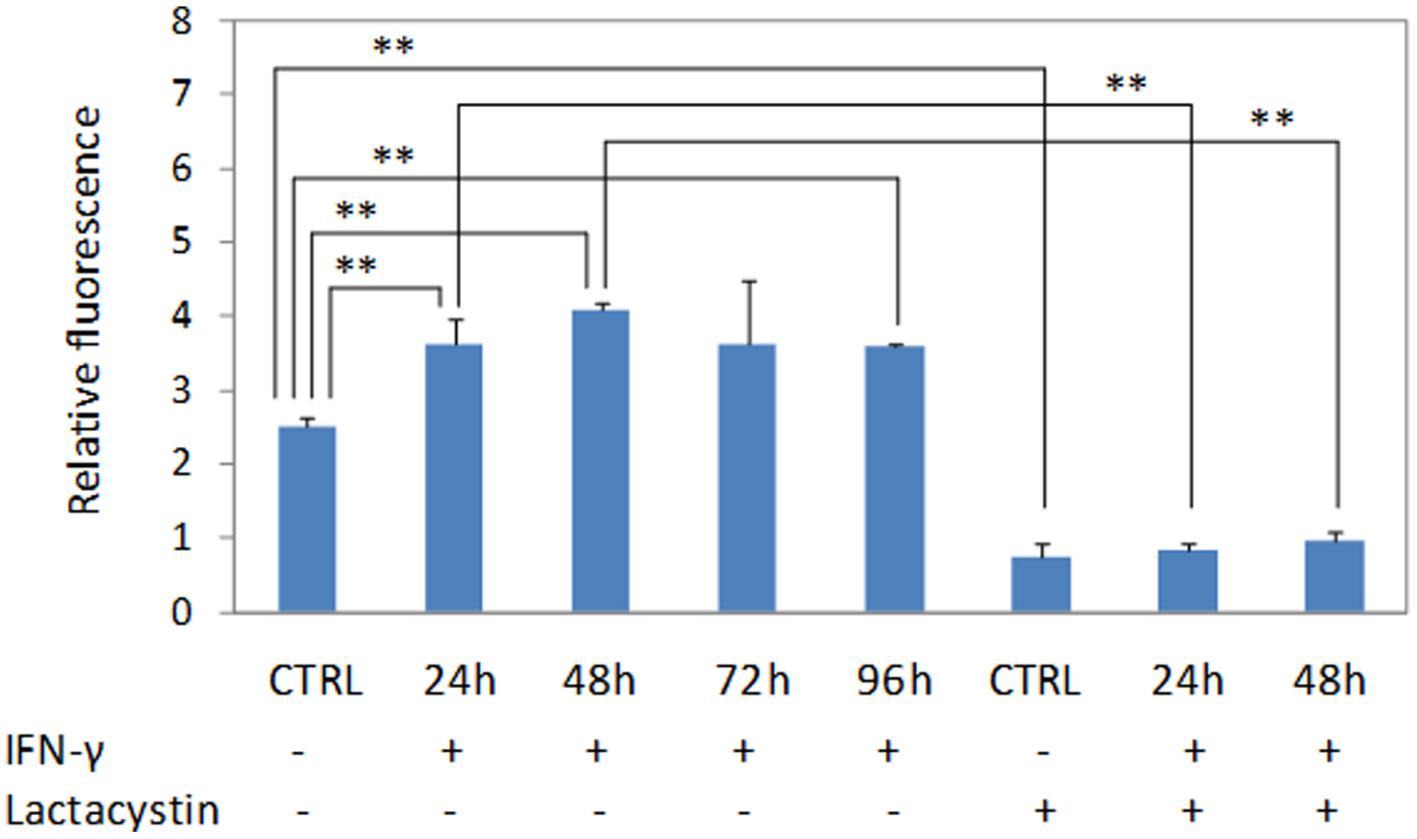
IFN-γ exposure increases immunoproteasome activities in HSG cells. The cells were treated with IFN-γ (250 U/ml) for 24–96 hrs, lysed with triton X-100, and then subjected to proteasome activity assay using fluorogenic substrate, Suc-LLVY-AMC. At each experimental condition, three cell lysates samples obtained from three separate T25 flasks (n = 3) were analyzed. **, p<0.01.

As shown in **Figure 4**, IFN-γ exposure increased immunoproteasome activities by 42–64% in HSG cells when the cells were treated with IFN-γ (250 U/ml) for 24–96 hrs. However, when the cells were pre-treated with lactacystin, the IFN-γ-induced immunoproteasome activities were significantly suppressed.

## Discussion

SS is an autoimmune disease in which dry eyes (keratoconjunctivitis sicca) and dry mouth (xerostomia) result from lymphocytic infiltration of lacrimal and salivary glands. During the formation and proliferation of the lymphocytic infiltrates in SS patients, IFN-γ is significantly over-produced in salivary glands and body fluids. For instance, both saliva and serum IFN-γ levels are 10-fold higher in SS patients than healthy individuals [11], [12]. In fact, salivary gland CD4+ T cells from patients with SS produce over 40-fold more IFN-γ mRNA than those from normal controls or the peripheral blood CD4+ T cells from the same patient. IFN-γ plays a critical role not only during the later immune phase of SS, but also in the early pre-immune phase, independent of effector functions of immune cells. Compared to NOD mice, both IFN-γ and IFN-γR gene knockout NOD mice (NOD.IFN-γ^-/-^ & NOD.IFN-γR^-/-^) showed no subsequent autoimmune response against the salivary glands [16]. IFN-γ at a relatively high concentration of 1000 U/ml causes anti-Fas mediated apoptosis by inducing Fas at the surface of salivary gland epithelial cells [13]–[15].

Our studies, however, suggest that IFN-γ may play a role in SS pathogenesis by inducing immunoproteasome activity in salivary gland cells. We have found that IFN-γ, at a relatively low level of 250 U/ml, induces the expression of β1i, β2i and β5i but strongly inhibits the expression of β1 and β5 in HSG cells. Constitutive β1, β2 and β5 subunits are endogenously expressed in HSG cells, forming constitutive proteasomes. However, under the stimulation of IFN-γ, β1, β2 and β5 are replaced by their βi counterparts, β1i, β2i and β5i, leading to the formation of immunoproteasomes and significantly increased immunoproteasome activities in HSG cells (Figure 4). Thus, immunoproteasome becomes one of dominant protein degradation mechanisms in IFN-γ-treated HSG cells. We will confirm these findings in our future studies. MS-based quantitative method can be used to further validate the altered expression of constitutive β1, β2 and β5 as well as immunoproteasome subunits β1i, β2i and β5i after the HSG cells are treated with IFN-γ. Similar experiments can be performed to study if IFN-γ induces immunoproteasome activities in primarily cultured salivary gland cells, and two-dimensional gel electrophoresis with MS may be used to investigate global protein changes altered by IFN-γ stimulation.

All the β subunits except β2 are synthesized as precursor proteins with an N-terminal propeptide sequence. The C-termini of beta subunits are on the outer surface of the proteasome whereas the N-terminal proteolytic active sites are in the inner surface. Upon assembling, the propeptides on the N-termini are removed by an autocatalytic mechanism and thereby the β subunits become catalytically activated [21]. The propeptides function to inhibit premature proteolytic activity and serve as chaperones to direct the proper proteasome assembly. They also protect the mature subunits catalytic threonine 1 alpha-amino group from acetylation [22]. In HSG cells, β1i, β2i and β5i are present mainly in mature forms (21, 24 and 23 kDa, respectively). As for the constitutive β subunits, β1 (24 kDa) and β5 (22 kDa) are mainly expressed as the mature forms and there is no propeptide sequence for β2.

In cells, constitutive proteasomes assemble first as half-proteasomes consisting of an alpha and beta ring, with the β2 subunit binding first to the alpha subunits followed by β1 and lastly β5 [23]. Two half proteasomes come together, which triggers the autolysis of the propeptide sequences and the degradation of proteassemblin, a chaperone [21], [24]. A complete 20S proteasome with 28 subunits is then formed, and the proteasome activator PA 700 binds to both ends of the 20S proteasome to allow entry of protein for degradation. Although β2 expression is not significantly affected by IFN-γ in HSG cells, the assembly of constitutive proteasomes can be diminished due to the expression of β1 is abolished under IFN-γ stimulation. Immunoproteasomes, however, assemble in a different order from constitutive proteasomes. β1i subunit is incorporated first to the alpha subunits and β2i subunit is then recruited. The incorporation of β2i into the proteasome actually depends on the expression of β1i. In the presence of β1i and β2i, the β5i subunit is incorporated last for the maturation of the immunoproteasome [6]. Due to dramatically increased expression of β1i, β2i and β5i in IFN-γ treated HSG cells, the assembly of immunoproteasomes is significantly enhanced and may reach the highest level when the HSG cells are treated with IFN-γ for 48 hours (Figures 1 & 4).

IFN-γ binds to its receptor on cell surface and activates the JAK-STAT pathway which induces the transcription of many genes. IFN-γR is indeed present on the ductal and acinar epithelia of normal human salivary gland tissue [13], [25]. Upon IFN-γ stimulation, many proteins may be altered in HSG cells due to the activation of JAK/STAT pathway. Meanwhile, because IFN-γ activates immunoproteasomes in HSG cells, ubiquitinylated proteins may be degraded by immunoproteasomes resulting in decreased levels in HSG cells. Our studies have demonstrated that IFN-γ not only induces the expression of the immunoproteasome β subunits, but also up-regulates the expression of MHC I in HSG cells. This may be due to that β1i, β5i and MHC I share similar promoter region [6], [7], [26]. β2i is located on chromosome 16 at p22.1 whereas β1i and β5i genes are mapped to p21.3 of human chromosome 6, the same region that contains the gene for MHC I.

Based on LC-MS/MS analysis, we found MHC class I on both untreated and IFN-γ-treated HSG cells whereas B2M, an important component of MHC I complex, was only present on IFN-γ-treated HSG cells (**Table 2**). This not only indicates that we successfully isolated the MHC I complex from the HSG cells through the Co-IP approach, but also suggest that B2M is up-regulated by IFN-γ in HSG cells. We previously discovered that the salivary levels of both B2M mRNA and protein are significantly higher in the patients with SS than healthy individuals. Further validation of B2M protein in a new SS patient cohort confirmed that it is a highly sensitive biomarker for SS [27], [28].

Immunoproteasomes efficiently produce antigenic peptides for MHC I presentation. Our studies suggested that potential MHC I peptides are presented on HSG cells due to IFN-γ stimulation. Although these peptides need to be further confirmed as MHC I-associated, several of them seem to have high binding affinity to MHC I according to the NetMHC-3.0 MHC-I binding prediction tool [29]. Autoimmune disease occurs when a specific adaptive immune response is mounted against self antigens. Autoreactive T-cells may be activated by MHC presentation of self-antigens, leading to chronic inflammatory damage to tissues. As a result of IFN-γ-mediated activation of immunoproteasomes in HSG cells, the peptides generated may be transported into endoplasmic reticulum where they bind to the MHC I complex and then presented on the cell surface. Autoreactive T cells are able to bind to the peptide-MHC I complex and trigger an immune response if the T cells recognize the peptides as non-self, causing the destruction of salivary gland cells in patients with SS.

We found lactacystin strongly suppressed the expression of β1i but did not significantly alter the expression of β5i and β2i in IFN-γ-treated cells. This might be because lactacystin plays an inhibitory role of the transcriptional regulation of β1i. Previous studies have indeed shown that lactacystin selectively reduces the expression of surface protein B via down-regulating thyroid transcription factor-1 [30]. β1i subunit is important for immunoproteasome assembly because it is the first β subunit that binds to the alpha subunits. In fact, the bound β1i subunit recruits β2i and β5i to assemble an immunoproteasome [6]. Our findings imply that lactacystin inhibits the formation of immunoproteasomes in HSG cells via suppressing the expression of β1i.

Our study also shows that the expression levels of all constitutive proteasome β subunits are not significantly affected by IFN-γ if HSG cells are pre-treated with lactacystin. On the other hand, IFN-γ treatment remains to strongly induce the expression of β2i and β5i even though HSG cells are pretreated with lactacystin. More importantly, lactacystin pretreatment strongly blocks the induced expression of β1i by IFN-γ in HSG cells.

In HSG cells without IFN-γ treatment, the expression of β1 was dramatically inhibited but β2 and β5 subunits were not significantly affected by lactacystin. To form a 20S constitutive proteasome, β2 subunit binds to the alpha subunits first; this is followed by the binding of β1 and β5 subunits [6]. Therefore, lactacystin also has an inhibitory effect on the proteasome formation in HSG cells due to dramatic down-regulation of β1.

Previous studies concluded that lactacystin functions by binding to the N-termini of the β subunits therefore inhibiting the formation of proteasomes [31], [32]. However, our studies suggest that lactacystin may inhibit the formation of proteasomes or immunoproteasomes in HSG cells by suppressing the expression of β1 or β1i subunits.

In summary, our studies have demonstrated that IFN-γ induces the expression of immunoproteasome β subunits and immunoproteasome activity but suppresses the expression of constitutive proteasome β subunits in HSG cells. Tandem MS analysis has allowed us to identify peptides possibly presented by MHC class I complex on HSG cells. In addition, lactacystin was found to suppress the expression of β1 subunit and significantly block IFN-γ-induced expression of β1i and immunoproteasome activity in the HSG cells. These results may add new insight into the mechanism regarding how proteasome inhibitors block the action of immunoproteasomes.

Our findings suggest a possible molecular mechanism underlying SS pathogenesis. As a result of IFN-γ over-expression in patients with early SS onset, immunoproteasomes are activated in salivary gland cells, leading to MHC I associated peptides presented on salivary gland cells. These peptide-presenting cells may be recognized and targeted by autoreactive T cells, causing the destruction of salivary gland cells in patients with SS. If this mechanism is further proven in human and mouse disease models, treatment of SS or slow-down of SS progression in the patients might be possible by targeting the peptide epitope(s) on HSG cells or using proteasome inhibitors to suppress immunoproteasomes. On the other hand, apoptosis of autoreactive T cells may be selectively induced, even in advanced autoimmune disease, by the introduction of the MHC class I and self-peptide complexes [33].

## Materials and Methods

### Cell culture and treatment

HSG cell line was initially established by Professor M. Sato of Tokushima University in Japan [34]. The cells were cultured in DMEM/F12 media supplemented with 10% fetal bovine serum (Gemini) and 1% penicillin-streptomycin (Gibco). All cells were maintained in a 5% CO_2_ incubator at 37°C.

HSG cells were treated with IFN-γ at a concentration of 250 U/ml for 24, 48, 72 or 96 hours. For lactacystin studies, HSG cells were pre-treated with 10 uM lactacystin for 1 hour and then treated with IFN-γ for 48 hours to investigate if lactacystin blocks the effect of IFN-γ stimulation.

### Western blotting

To prepare cell lysates for Western blot analysis, HSG cells were harvested and lysed with the 2-DE rehydration buffer. Total protein assay was performed using the 2-D Quant Kit from GE Healthcare.

Cell lysates (30 µg total proteins of each sample) were separated with a 12% NuPAGE gel (Invitrogen) at 120 V for approximately 90 min and then transferred to nitrocellulose membrane using the iBlot system (Invitrogen). Membranes were blocked with 5% milk in TBST buffer for 2 hours and then incubated in a primary antibody (Abcam: mouse monoclonal antibody to β1i, ab78336; mouse monoclonal antibody to β2i, ab77735; mouse monoclonal antibody to β5i, ab58094; mouse monoclonal antibody to β1, ab21809; mouse monoclonal antibody to β2, ab22650; rabbit polyclonal antibody to β5, ab78140; Thermo Fisher: mouse monoclonal antibody to MHC I, W6/32, MA1-19027) overnight at 4°C. Following incubation with secondary antibody horseradish peroxidase-conjugated mouse or rabbit antibody (Applied Biological Materials, Canada), protein bands were detected using the ECL Kit (GE Healthcare). Scanned images were quantified with ImageJ (NIH) and ANOVA analysis (Tukey's Post Hoc test) was used to determine statistical differences between different treatment groups.

### Isolation and analysis of MHC I complex

M-280 tosylactivated Dynabeads (Invitrogen) were first washed in 0.1 M borate buffer for five minutes while shaking. For the coating procedure, the beads were incubated with W6/32 antibody, which recognizes the MHC class I when bound to β2 M and peptide, for 36 hours at room temperature. After incubation, the coated beads were washed twice in PBS/0.1% BSA (pH 7.4) for 5 minutes at 4°C. The beads were then incubated in 0.2 M Tris/0.2% BSA (pH 8.5) for 24 hours at room temperature. Finally, the beads were washed twice in PBS/0.1% BSA (pH 7.4) for 5 minutes at 4°C.

Cells were collected using a Cell Stripper (Cellgro, Mediatech) and washed extensively with PBS, followed by re-suspension in 80 mM Na_2_HPO_4_ (pH 8.0). Cells were treated with papain (0.125 mg/ml, Sigma) at 37°C in CO_2_ incubator for 90 minutes. Following digestion, the cells were centrifuged at 13,000 g for 30 minutes at 4°C. The supernatant was concentrated using Amicon Ultra 30K membrane (Millipore) at 500 g for 2 hours at 4°C. The retentate containing MHC class I was added to the Dynabeads coated with W6/32 monoclonal antibody for 3-hour incubation, followed by extensive washes with PBS. Trifluoroacetic acid at pH 2.1 was added to the beads to elute the bound MHC I, beta-2-microglobulin (B2M) and peptide complex. The recovered effluent was purified with Amicon Ultra 3K filter at 13,000×g for 1 hour at 4°C. The filtrate containing the MHC class I peptides was subjected to liquid chromatography with tandem mass spectrometry (LC-MS-MS) using a nano-LC system (Eksigent Technology) and LTQ ion trap mass spectrometer (Thermo Finnigan). LC separation of peptides was performed with C18 PicoFrit capillary columns (New Objectives) at a flow rate of 400 nL/min. Database search was performed against the SwissProt database using the SEQUEST (Thermo Finnigan). The following parameters were used for database searching: enzyme specification, semi-tryptic/semi-chymotryptic; missed cleavage allowed, one; Xcorr>2.0 (2+), Xcorr>2.5 (3+). Meanwhile, to identify proteins within MHC I complex, the retentate containing the MHC I complex proteins was first reduced with 10 mM DTT for 30 min, followed by alkylation with 50 mM iodoacetamide for 60 min in the dark, and then digested with 10-ng trypsin at 37°C overnight. The resulting peptides were identified by LC-MS/MS with database searching, as described above.

### Proteasome Activity Assay

After preparation of HSG cell lysates in 1.0% triton X-100 buffer, proteasome activities were measured using the Proteasome Activity Assay Kit (APT280, Millipore) and fluorogenic substrate Suc-LLVY-AMC according to the manufacturer's manual. The fluorescence was read with a fluorometer (Synergy HT Microplate Reader, BioTek Instruments), and the excitation and emission wavelengths were 380 and 460 nm, respectively.
